# Wide-band Beam-scanning by Surface Wave Confinement on Leaky Wave Holograms

**DOI:** 10.1038/s41598-019-49619-7

**Published:** 2019-09-13

**Authors:** Mohammad Moein Moeini, Homayoon Oraizi, Amrollah Amini, Vahid Nayyeri

**Affiliations:** 1 School of Electrical Engineering, Iran University of Science and Technology, 1684613114 Tehran, Iran; 2 Antenna and Microwave Research Laboratory and School of New Technologies, Iran University of Science and Technology, 1684613114 Tehran, Iran

**Keywords:** Electrical and electronic engineering, Electrical and electronic engineering, Condensed-matter physics, Condensed-matter physics

## Abstract

A two-dimensional (2-D) metasurface design for backward leaky wave suppression in microwave regime is proposed based on the theory of holography. The so-called Rabbit’s ears phenomenon describes that the backward mode in the reference wave plays the destructive role and makes the holography principle to behave properly mainly in an only narrow frequency interval. Here, we explore the utilization of the surface wave reflectors to suppress the backward mode to achieve wide-band holograms. Therefore, the reference wave form is manipulated by the choice of various reflector shapes and some providing forward mode dominant reference wave are analyzed and simulated. The less backward mode participates in the reference wave; the wider operation frequency range is obtained. With the canceled Rabbit’s ears phenomenon, variations in the reference wave frequency cause elevation angle scan. The results provide general insights into relation of the Rabbit’s ears phenomenon and the object wave accuracy in frequencies except the design frequency. The idea is also applied to multiple object wave holograms. The concept is verified using both electromagnetic full-wave simulations and experimental measurements.

## Introduction

Controlling electromagnetic waves by the aid of metamatrials and metasurfaces has recently paved the way of beam manipulation in a more modern way^1^. Metasurfaces provide the integrablity of the electronic components due to the planar shape. This brings degrees of freedom for wave direction^2–4^ and polarization manipulation^5^ and other certain applications like focusing^6^, wave routing^7^ and scattered wave engineering^8^.

Surface wave leakage manipulation is one of the important applications of the metasurfaces. In physics of electromagnetic wave, decrease in amplitude of propagating wave in a lossless medium is associated with energy leakage out of the propagation medium. The propagation medium can be waveguide with metallic boundary, dielectric slab, metasurface waveguide, spoof surface plasmon polariton (SSPP) waveguide and etc. Wave leakage can be brought into play by local perturbation in the propagation medium. The key advantage of the leaky wave systems is the ability of control on the amplitude and propagation direction of the leaky waves^9^. Holography principle for metasurface design is a powerful tool for wave maniplulation in any desired direction. The theory of optical holography was first described by Gabor^10^, and then the theory was suggested to be utilized in microwave regime by Chaccacci^11^. Leaky wave hologram capability was verified for pattern synthetization in a desired direction first by Sievenpiper^12^. Periodic grounded patches supporting *T* *M*_0_ surface wave are used to realize this concept. The possibility of polarization manipulation using proper surface impedance distribution has been verified and a new degree of freedom has been added to hologram designs using anisotropic unit-cells^13,14^. One of the key advantages of the leaky wave structures is the scan capability which can be implemented for Frequency Modulated Continuous Wave (FMCW) systems. Therefore, wide-band operation is a valuable property of leaky wave metasurfaces. However, for 2-D leaky wave metasurfaces designed using holography, pleasant beamforming can be achieved at a single frequency only. Some approaches have been suggested to improve the operating frequency in recent literature. For instance, an increase in bandwidth can be obtained by layering the substrate^15^. However, side lobe level (SLL) is not acceptable at higher frequencies. In another work, 1-D scanning angle from −19° to 12° at frequency interval 14–20 GHz is reported by Cui^16^. One solution is to locate the wave launcher at corner of the hologram^17^. Another is to sectorize the hologram for the launcher power conversion into multi-beam radiation^18^. Nevertheless, to achieve high beam directivity at a desired direction and low edge diffraction, utilization of 2-D center-fed leaky wave metasurfaces seems inevitable for practical implementation.

In this work, reflector-enabled 2-D holograms are proposed for wide-band beamforming and scannability. The presence of reflector on metasurface structures may seem to have priority from two point of views; the reflector may contribute in radiation characteristics enhancement, or in some cases, the presence of a reflector as an obstacle is inevitable like vehicular and satellite antennas. Lack of acceptable radiation directivity and bandwidth in center-fed hologram is caused due to a destructive effect called Rabbit’s ear phenomenon^17^. In 2-D holographic radiators, the radiation corresponding to the forward and backward modes play a constructive role only at design frequency. In fact, the scanning steps for the forward and backward modes are only equal at the design frequency. By placing a reflector on the hologram, backward mode distribution can be changed to eliminate the Rabbit’s ear phenomenon. The surface impedance distribution and the corresponding reference wave must be modified to scatter the desired object wave after placing the reflector. The modified impedance is depending on the geometry of the wave reflector placed on the hologram. Radiation characteristics and scannabality by frequency may also be enhanced even for multi-beam structures. We propose the surface wave confinement on holographic metasurfaces using surface scatterers which can cancel out the Rabbit’s ears phenomenon in a more beneficial way than the previous reflector-free works. As the following explorations prove, surface wave confinement on hologram causes considerable improved radiation bandwidth and scannability for single and multi-beam holograms. The proposed reflector-enabled hologram can achieve single-beam frequency scanning between 13–19 GHz (representing 37.5% fractional bandwidth) and multi-beam frequency scanning between 14–18 GHz (25% fractional bandwidth).

## The Theory of Holography in Microwave Regime

Based on the theory of holography, a desired wave profile is produced by exciting the hologram with needed excitation wave. The desired and excitation waves are called object wave (${\psi }_{obj}$) and reference wave (${\psi }_{ref}$), respectively. The recorded interference of reference and object waves called interferogram can project the object wave while it is excited by the reference wave^19,20^. The concept has laid the foundations for holographic wave manipulation in both optical and microwave regimes. Metasurfaces emulate the interferograms holographic application in microwave. Leaky wave holographic metasurfaces are formed from quasi-periodic surface impedance. In this case, the surface impedance needed for object wave production is defined as

1 $${Z}_{s}=j{X}_{0}{\eta }_{0}(1+M\times \Re \{{\psi }_{ref}{\psi }_{obj}^{\ast }\})$$

where *X*_0_ and *M* represent normalized impedance with respect to the free-space impedance $({\eta }_{0})$ and modulation factor, respectively. $\Re$ denotes the real part of a complex value. The reference wave can be chosen as whether space-propagating or surface wave. The reference wave for our desired application is defined as a surface wave in a form of Hankel function of the second kind as

2 $${\psi }_{ref}={a}_{0}{H}_{0}^{(2)}(\beta \sqrt{{x}^{2}+{y}^{2}})$$

where *β* is the phase constant for the *T* *M*_0_ surface mode and is obtained as

3 $$\beta ={k}_{0}\sqrt{1+{X}_{0}^{2}}$$

Note that the value of *β* is an approximation of the wave number which is utilized for the object wave calculation and derivation of the associated surface impedance. The approximated value of *β* is valid when *M* is small enough^21^. In case that the modulation depth is not small, *β* should be calculated using the dispersion relation which will be proposed later in this paper. The object wave of interest in this work is a plane wave propagating in a certain direction as

4 $${\psi }_{obj}={e}^{-j(kx\sin {\theta }_{0}\cos {\varphi }_{0}+ky\sin {\theta }_{0}\sin {\varphi }_{0})}$$

**Figure 1:**
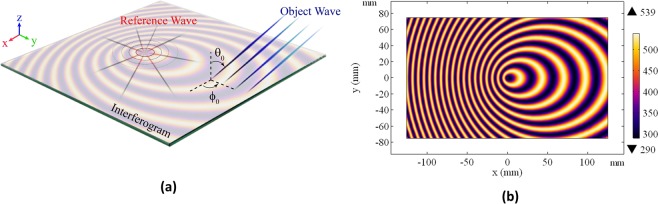
(**a**) Representation of the reference and object waves. (**b**) The holographic interference pattern.

Figure 1a represents a typical radiative metasurface using holographic surface impedance (interferogram) for beamforming. Here *θ*_0_ and ${\varphi }_{0}$ are the spherical angles for the object wave propagation direction. The hologram uses cylindrical surface wave to excite the surface impedance to form the object wave. The precise knowledge of 2-D surface impedance distribution is crucial for accurate holographic beamforming. For instance, Fig. 1b shows a 2-D surface impedance distribution aimed to produce an object wave at ${\theta }_{0}=60^\circ$ and ${\varphi }_{0}=90^\circ$ while it is excited by the reference wave given in (2). The surface impedance is calculated using (1). The color bar on the right shows the imaginary value of the surface impedance for the sample from minimum to maximum.

### Rabbit’s Ears Phenomenon in Holographic Metasurfaces

In this section, we start the holographic analysis by a center-fed mono-dimensional hologram to illustrate the situation. The reference and object waves are chosen as ${\psi }_{ref}={e}^{-j\beta |x|}$ and ${\psi }_{obj}={e}^{-j{k}_{0}\sin {\theta }_{0}x}$, where *β* is the real part of the fundamental wave number tangential to the surface impedance $\kappa$ ($\kappa =\beta -j\alpha$) and *α* is the attenuation factor along the leaky wave metasurface. In this case, the reference wave on positive and negative directions of x axis are called forward and backward surface leaky modes, respectively. According to (1), the surface impedance *Z*_*s*_ can be expressed as

5 $${Z}_{s}=j{X}_{0}(1+M\,\cos (\beta |x|-{k}_{0}\,\sin \,{\theta }_{0}x))$$

For this sinusoidally-modulated surface impedance, the periodicity is

6 $$d=\frac{2\pi }{\beta -\frac{x}{|x|}{k}_{0}\,\sin \,{\theta }_{0}}$$

To specify *d*, the expression for *β* should be known. The dispersion equation can be written in continued fraction form^22^:

7 $$\begin{array}{rcl}1-\frac{j{\eta }_{0}}{{X}_{0}}\sqrt{1\,-\,{(\frac{\kappa }{{k}_{0}})}^{2}} & = & \frac{{M}^{2}}{4}\{\frac{\,\,\,\,\,\,1\,\,\,\,\,\,|}{1-\frac{j{\eta }_{0}}{{X}_{0}}\sqrt{1-{(\frac{\kappa }{{k}_{0}}+\frac{2\pi (-1)}{{k}_{0}a})}^{2}}}\\  &  & -\,\frac{\,\,\,\,\,\,{M}^{2}/4\,\,\,\,\,\,|}{|1-\frac{j{\eta }_{0}}{{X}_{0}}\sqrt{1-{(\frac{\kappa }{{k}_{0}}+\frac{2\pi (-2)}{{k}_{0}a})}^{2}}\,\,\,\,\,\,}-\ldots \\  &  & +\,\frac{\,\,\,\,\,\,1\,\,\,\,\,\,|}{1-\frac{j{\eta }_{0}}{{X}_{0}}\sqrt{1-{(\frac{\kappa }{{k}_{0}}+\frac{2\pi (1)}{{k}_{0}a})}^{2}}}\\  &  & -\,\frac{\,\,\,\,\,\,{M}^{2}/4\,\,\,\,\,\,|}{|1-\frac{j{\eta }_{0}}{{X}_{0}}\sqrt{1-{(\frac{\kappa }{{k}_{0}}+\frac{2\pi (2)}{{k}_{0}a})}^{2}}\,\,\,\,\,\,}-\ldots \}\end{array}$$

**Figure 2:**
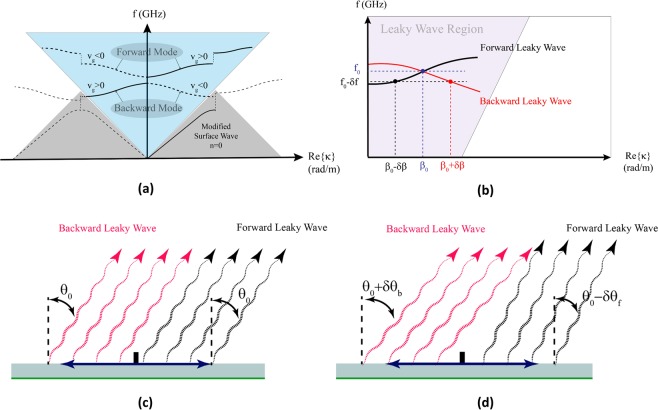
Comparison of the beam angles due to the forward and backward modes on a symmetrical modulated surface impedance. (**a**) Typical dispersion diagram of the 1-D leaky wave structure for periodic small perturbations. (**b**) Dispersion comparison between the forward and backward leaky wave modes at the design frequency (*f*_0_) and a slightly lower than the design frequency sample $({f}_{0}-\delta f)$ on an asymmetrical surface impedance. Propagation directions illustration of forward and backward leaky waves at (**c**) *f*_0_ and (**d**) ${f}_{0}-\delta f$ operation frequencies.

The typical dispersion diagram of the forward and backward surface wave phase constants for a symmetrical surface impedance (equal periods on forward and backward regions) is shown in Fig. 2a. The diagram indicates that a center-fed leaky wave metasurface with equal surface impedance periods for forward and backward regions is not capable of beamforming at a single direction because the leaky waves from forward and backward surface waves propagating at different directions.

Equation (6) shows that for a leaky wave metasurface designed to radiate toward a direction in the upper half-space ($0^\circ  < {\theta }_{0} < 90^\circ$), the period of the modulated surface impedance is shorter on the backward leaky mode region relative to the forward leaky mode region. Figure 2b represents the dispersion diagram for a center-fed leaky wave 1-D surface impedance designed to produce a tilted object wave with respect to the normal vector to the metasurface plane using (5). Considering the asymmetrical surface impedance distribution on each side of the surface wave feed, as it is shown in Fig. 2b, the dispersion curves corresponding to each forward and backward modes cross at *β*_0_ which is associated with design frequency *f*_0_. In other words, only identical *β* for the forward and backward leaky waves make the radiated object waves to participate in a constructive manner by propagation in a single direction. Therefore, to design a conventional bilateral hologram, it is necessary for the forward and backward regions surface impedance periods to be chosen in a way that the dispersion curves cross each other at (*f*_0_, *β*_0_). Any variation in frequency makes the *β*_*f*_ (forward leaky wave phase constant) and *β*_*b*_ (backward leaky wave phase constant) to separate from *β*_0_ on the dispersion diagram. Therefore, the *θ*_*f*_ and *θ*_*b*_ deviate from each other. At frequency ${f}_{0}-\delta f$, the forward and backward modes’ angles with respect to the normal direction are ${\theta }_{f}-\delta {\theta }_{f}$ and ${\theta }_{b}+\delta {\theta }_{b}$, respectively. Thus, at any other frequency except *f*_0_, the total radiation pattern is not acceptable. This destructive effect on the object wave is called Rabbit’s ears phenomenon^17^. Figure 2c,d illustrate the operation frequency restriction of radiative leaky wave antennas. They show the leaky wave directions of forward and backward modes at the design frequency *f*_0_ and ${f}_{0}-\delta f$.

**Figure 3:**
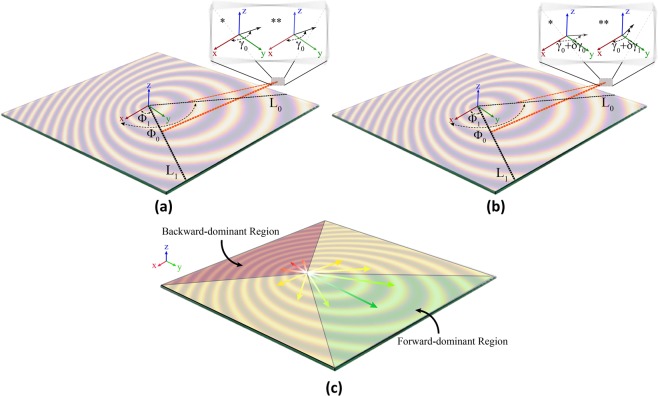
(**a**) Hologram operation at *f*_0_, (**b**) Hologram operation at ${f}_{0}-\delta f$. The single star (*) is for the beam generated by surface wave along *L*_0_ and double star (**) is for the beam generated by surface wave along *L*_1_. (**c**) The regions on the metasurface including the forward-dominant and backward-dominant regions for a tilted object wave generation using a center-fed cylindrical surface wave launcher.

This concept may be generalized to a 2-D hologram (see Fig. 3). Assuming the object wave as a pencil beam directed in *γ*_0_ with respect to x axis at *f*_0_, two hypothetical lines *L*_0_ and *L*_1_ are projected on the metasurface which are located at ${\Phi }_{0}$ and ${\Phi }_{1}$, respectively. Unit-cells (realized surface impedance) on the metasurface which are along *L*_0_ and *L*_1_ both contribute to produce the object wave in *γ*_0_ direction at *f*_0_. If the frequency is shifted from *f*_0_, the scanning direction and surface impedance step corresponding to the unit-cells along *L*_0_ and *L*_1_ will be different from each other. Hence, at ${f}_{0}-\delta f$, the leaky waves produced by the unit-cells along *L*_0_ and *L*_1_ propagate in different directions. Therefore, the Rabbit’s ears phenomenon occurs and beamforming at a single direction is impossible when frequency is shifted from the desired frequency *f*_0_ (Fig. 3b).

It can be concluded from the above discussion that for a distortion-free radiation pattern over a frequency interval, a backward mode suppression is essential. Apart from mathematics, for a hologram aimed to generate a tilted object wave through Y-Z plane, an overall deduction can be generalized from Fig. 3c that the more surface wave vectors seem alike with a y-directed surface wave, the less destructive effect occurs. In other words, the green arrows represent the most constructive case, while the red ones represent the most destructive case. We propose confinement of the surface wave using reflectors. Therefore, a proper selection of surface wave reflector geometry may cause surface wave confinement to the forward-dominant region. The surface wave confinement into forward mode radiating section eliminates the backward mode destructive effect.

## Leak Wave Holography in Presence of Surface Wave Reflector

### Single Object Wave

Monopole launcher on a grounded dielectric is a proper choice for *T* *M*_0_ surface wave production. The electric and magnetic fields of the monopole launcher with moment *I*_0_Δ*z* in a cylindrical coordinate can be written as^13^

8 $${\overrightarrow{E}}_{sw}=[j{X}_{s}{J}_{sw}{H}_{1}^{(2)}(\beta \rho )\hat{\rho }+{J}_{sw}\frac{\beta {\eta }_{0}}{{k}_{0}}{H}_{0}^{(2)}(\beta \rho )\hat{z}]\,{e}^{-{\alpha }_{z}z}$$

9 $${\overrightarrow{H}}_{sw}=-\,{J}_{sw}{H}_{1}^{(2)}(\beta \rho )\,{e}^{-{\alpha }_{z}z}\hat{\varphi }$$

where *β* and *α*_*z*_ are the phase constant in the propagation direction and the attenuation constant in the *z* direction, respectively. *X*_*s*_ is the positive surface reactance of the grounded dielectric and *J*_*sw*_ is defined^23^

10 $${J}_{sw}=-\,{I}_{0}\Delta z\frac{{k}_{0}\beta {X}_{s}}{2}$$

With the aid of the Poynting’s vector, the power carrying by above fields is

11 $$\begin{array}{rcl}{\overrightarrow{S}}_{sw} & = & {\overrightarrow{E}}_{sw}\times {{\overrightarrow{H}}_{sw}}^{\ast }\\  & = & -\,j{X}_{s}|{J}_{sw}{|}^{2}|{H}_{1}^{(2)}(\beta \rho ){|}^{2}{e}^{-2{\alpha }_{z}z}\hat{z}\\  &  & +\,\frac{{\eta }_{0}\beta }{{k}_{0}}|{J}_{sw}{|}^{2}{H}_{0}^{(2)}(\beta \rho ){H}_{1}^{(2)\ast }(\beta \rho ){e}^{-j2{\alpha }_{z}z}\hat{\rho }\end{array}$$

The *z* component of the Poynting’s vector is pure imaginary demonstrating that the power does not leave the dielectric slab along the *z* direction, i.e. the power density only surges up and down with no propagation^24^. In other words, the *z*-directed power density is stored and the propagation occurs only in $\rho$ direction. The imaginary part of the Poynting’s vector exist at $\rho$ and *z* directions which represents the coupling of the wave to the dielectric. The real part also explains wave propagation in $\rho$ direction and this part only impresses contribution in holography equation (1). Therefore, the reference wave can properly be designated as zero order Hankel function of the second kind.

Let us consider an infinitely long conducting wire within a medium with refractive index $n=\sqrt{1+{X}_{0}^{2}}$. If the wire is directed along the *z* axis, the radiated electric and magnetic fields are in the *z* and $\varphi$ directions, respectively and can be written as^25^

12 $${E}_{z}=-\,{I}_{e}\frac{\omega \mu }{4}{H}_{0}^{(2)}(\beta \rho )$$

13 $${H}_{\varphi }=-\,j{I}_{e}\frac{\beta }{4}{H}_{1}^{(2)}(\beta \rho )$$

where *I*_*e*_ is a constant. Comparing (12) with (8) and (13) with (9), it can be concluded that the monopole wave launcher and long conducting wire represent an equivalent wave function at $z=0$ on the metasurface.

As mentioned before, the Rabbit’s ears phenomenon leads to a destructive effect on the radiation characteristics of the hologram. To prevent this destruction, we propose using a PEC wall reflector. It can be predicted that the scattering effect of the reflector changes the destructive backward mode into a constructive forward mode which results in improving the radiation characteristics. However, looking back to Fig. 3c, to cancel out the Rabbit’s ears phenomenon in 2-D, wedge reflector as a surface wave reflector seems more appropriate. To verify this idea, we need the surface wave function of the cylindrical wave launcher next to the wedge reflector which can be derived analytically. For an infinite conducting wire placed next to a wedge reflector at ${\rho }_{0}$ and ${\varphi }_{0}$, the total wave expression can be written as^25^

14 $${\psi }_{ref}=(\begin{array}{ll}\sum _{\nu }\,{a}_{\nu }{J}_{\nu }(k\rho ){H}_{\nu }^{(2)}(k{\rho }_{0})\,\sin \,(\nu (\varphi -{\varphi }_{0}))\,\sin \,(\nu (\varphi -\zeta )) & \rho  < {\rho }_{0}\\ \sum _{\nu }\,{a}_{\nu }{J}_{\nu }(k{\rho }_{0}){H}_{\nu }^{(2)}(k\rho )\,\sin \,(\nu (\varphi -{\varphi }_{0}))\,\sin \,(\nu (\varphi -\zeta )) & \rho  > {\rho }_{0}\end{array}$$

where $k=n{k}_{0}$, $\zeta$ is half of the total inner wedge angle, and ${J}_{\nu }$ represents the Bessel function of the first kind of order $\nu$. Satisfying boundary conditions on the wedge and conducting wire will result $\nu$ and ${a}_{\nu }$ as

15 $$\nu =\frac{m\pi }{2(\pi -\alpha )}\,m=1,2,3,\ldots $$

16 $${a}_{\nu }=-\,\frac{\pi \omega \mu {I}_{e}}{2(\pi -\alpha )}$$

With the modified reference wave, the modified surface impedance *Z*_*s*_ must be calculated using holography theorem. The surface impedance is obtained by substituting the reference wave expression (14) into (1) and then the object wave is produced when the newly resulted interferogram is excited by the modified reference wave.

**Figure 4:**
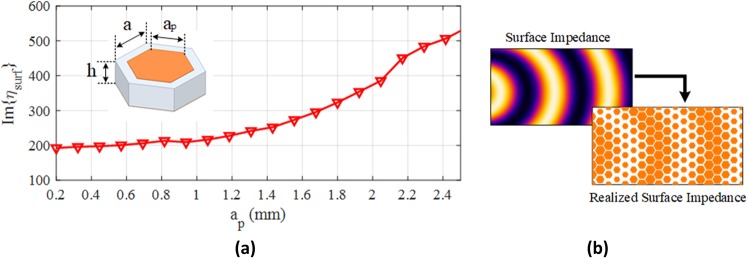
Realization process. (**a**) Imaginary part of surface impedance for hexagonal metallic patch side lengths. The results are for *a* = 2.7 mm. (**b**) Assigning different surface impedance values to corresponding metallic patches.

The surface impedance realization requires a unit-cell design to represent the corresponding surface impedance cell obtained from surface impedance discretization. The unit-cell dimensions must be chosen relatively smaller than the operating wavelength of the hologram. Hexagonal unit-cell is proposed for surface impedance realization. The unit-cell with hexagonal lattice shape is more well-behaved considering the dispersion isotropy^26,27^. Figure 4a shows the hexagonal unit-cell and the surface impedance variations versus the length of the hexagon side (*a*_*p*_). The surface impedance is calculated using the equivalent circuit method^21^. Figure 4b shows how the surface impedance is realized by the hexagonal unit-cells.

**Figure 5:**
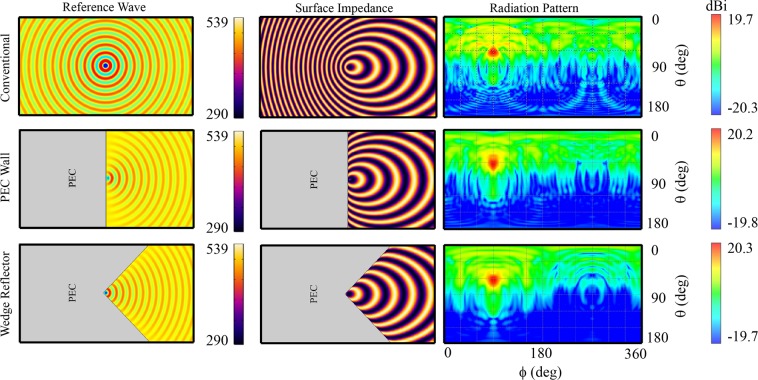
Comparison of reference waves, surface impedances, and 2-D distributed radiation patterns of conventional and reflector-enabled (PEC wall and wedge reflector) holograms all aimed to produce a certain object wave (${\varphi }_{0}=90^\circ$ and ${\theta }_{0}=60^\circ$). The simulation results are for the design frequency *f*_0_ = 18 GHz.

Three different leaky wave hologram configurations are compared: conventional hologram (without reflector), a hologram with a PEC wall reflector, and a hologram with a wedge PEC reflector. Figure 5 shows the reference wave, the surface impedance, and the 2-D distributed pattern of each. The surface impedances and 2-D patterns are resulted for the purpose of generating an object wave in ${\varphi }_{0}=0^\circ$ and ${\theta }_{0}=60^\circ$ direction at ${f}_{0}=18\,{\rm{GHz}}$. The directivity for the conventional hologram is obtained 18.3 dB, while for the holograms with PEC wall and wedge reflector the results are 20.2 dB and 20.3 dB at the design frequency 18 GHz, respectively. A relatively low directivity of the conventional hologram at the design frequency *f*_0_ can be attributed to the lack of enough approximation for the shorter surface impedance periods on the backward region of the conventional hologram. The rapid surface impedance variations on the backward region require higher 2-D discretization rate relative to the surface impedance on the forward region. Therefore, conventional leaky wave hologram shows high fabrication demands for realizing surface impedance on the backward region. The backward surface waves in reflector-enabled structures are redirected into forward region. The surface impedance on the reflector-enabled holograms are made of longer periods compared to the backward region of conventional hologram, as seen in Fig. 5. Thus, an advantage of the proposed reflector-enabled leaky wave holograms is simpler fabrication necessities for realization of unit-cell by elimination of very small unit-cells in the backward region.

Apart from the design frequency *f*_0_, a superior functional advantage of the reflector-enabled leaky wave holograms occurs. Comparing Figs 3c and 5, the hologram with wedge reflector seems to confine the surface waves into forward-dominant region more efficiently and less Rabbit’s ears phenomenon is predicted for this structure when the operating frequency is shifted from *f*_0_. It is known that at frequencies other than *f*_0_, the backward and forward leaky waves directions deviate. However, there is no backward leaky wave for the hologram with wedge reflector and all the reflected surface wave contribute to object wave construction as forward mode. The result is a single beam with directivity maintenance over a wide frequency range. The peak of the radiation pattern as a function of frequency is given approximately^22^

17 $${\theta }_{-1}(f)={\sin }^{-1}\,[\frac{\beta +\frac{2\pi (\,-\,1)}{d}}{{k}_{0}}]$$

where the subscript −1 indicates the dominant spatial harmonic in leaky wave radiators (mode *n* = −1). Therefore, frequency variation causes beam scanning.

**Figure 6:**
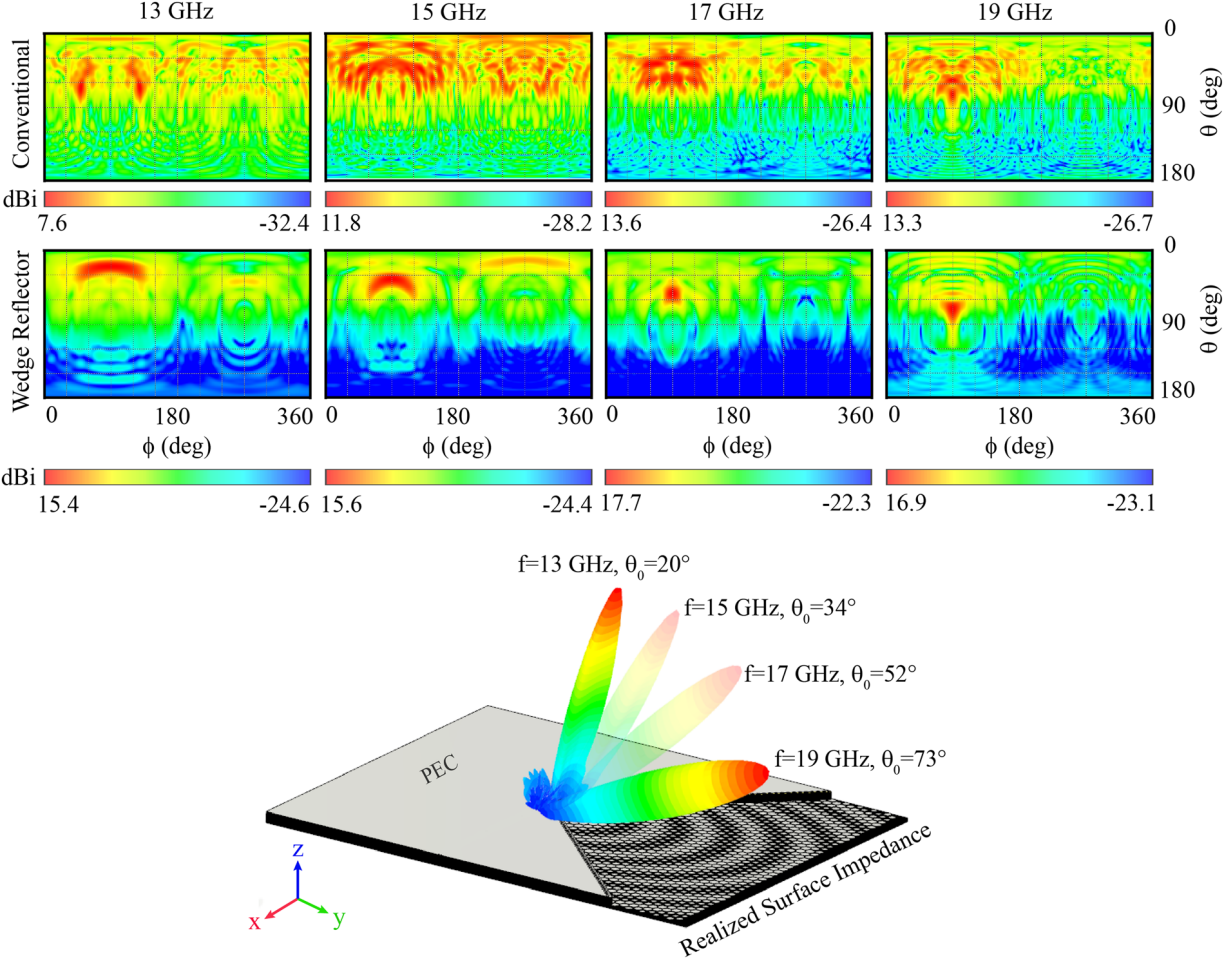
Pattern comparison of conventional hologram and the proposed hologram with wedge reflector. Illustration of the scannability property of the proposed hologram.

Figure 6 depicts the radiation patterns of the conventional and the proposed wedge reflector-supported holograms at different frequencies. Although, as shown in Fig. 5, the conventional hologram forms the desired beam at *f*_0_ = 18 GHz, as shown in Fig. 6, the beamforming is not acceptable at nearby frequencies, e.g., 13, 15, 17, and 19 GHz. On the other hand, by applying the proposed wedge reflector-supported hologram, an object wave is formed not only at the design frequency *f*_0_ = 18 GHz but also at a wide frequency range. The figure also shows that for the proposed hologram, the progressive variation of frequency changes the object wave radiation direction in a way that the hologram is capable of frequency scanning of elevation angle (*θ*) from 20° to 73° by varying the frequency over 13–19 GHz. The directivity for surface impedance modulated leaky wave antennas is^28^:

18 $$D\propto \frac{{\beta }_{\perp (-1)}}{{\alpha }_{\parallel (-1)}}=\frac{\sqrt{{\omega }^{2}{\mu }_{0}{\varepsilon }_{0}-{[\kappa +\frac{2\pi (-1)}{d}]}^{2}}}{{\alpha }_{\parallel (-1)}}$$

where subscript (−1), ${\beta }_{\perp }$ and ${\alpha }_{\parallel }$ indicate unbounded propagating mode number, phase constant in normal direction and attenuation constant in parallel direction relative to the metasurface plane, respectively. The modulation factor M determines the surface wave attenuation constant^14^. More values for M result more surface wave attenuation and therefore lower directivity. This means that for a forward surface wave leaky wave antenna, due to the nature of leaky wave antennas, the directivity will reduce as the beamforming angle gets close to the broadside angle. Hence, as may be observed from (18), lowering the modulation factor M causes smaller ${\alpha }_{\parallel }$. With weak attenuation constant, the leaky wave antenna requires longer propagation path for the desired radiation and larger antenna dimensions seem inevitable. Therefore, the trade-off between directivity and antenna dimensions should always be considered.

**Figure 7:**
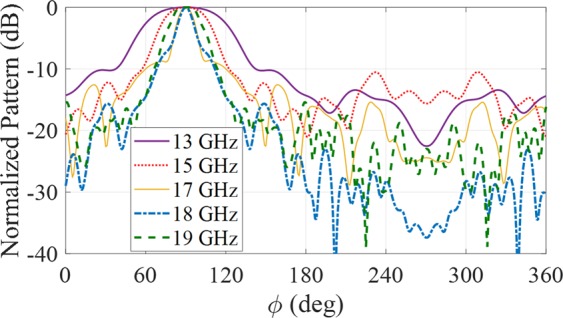
H-plane radiation pattern of wedge-reflector hologram at different frequencies.

In Fig. 7 H-pane patterns of the proposed wedge reflector-enabled antenna are compared from 13 to 19 GHz.

**Figure 8:**
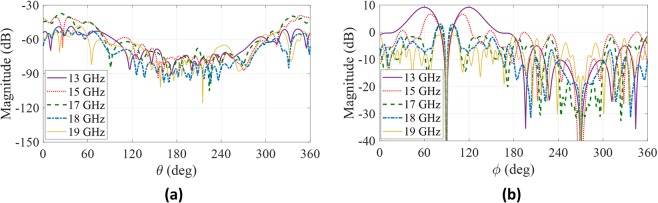
Cross-polarization patterns of the proposed antenna at different frequencies: (**a**) E-plane. (**b**) H-plane.

In general, the cross-polarization is low for this type of antenna both in E-plane and H-plane. Figure 8 shows the cross-polarization in E and H planes versus frequency. The cross-polarization is acceptable (less than −30 dB) at the design direction ${\theta }_{0}=60^\circ$ and ${\varphi }_{0}=90^\circ$. For other azimuth angles, the cross-polarization value is increased due to isotropic unit-cell^14^.

### Multiple Object Waves

Multi-beam generation by holography requires the summation of each individual object waves making up the total object wave expression. In order to generate object waves directed in (*θ*_1_, ${\varphi }_{1}$) and (*θ*_2_, ${\varphi }_{2}$), the object wave is defined

19 $${\psi }_{obj}={e}^{-j(kx\sin {\theta }_{1}\cos {\varphi }_{1}+ky\sin {\theta }_{1}\sin {\varphi }_{1})}+{e}^{-j(kx\sin {\theta }_{2}\cos {\varphi }_{2}+ky\sin {\theta }_{2}\sin {\varphi }_{2})}$$

**Figure 9:**
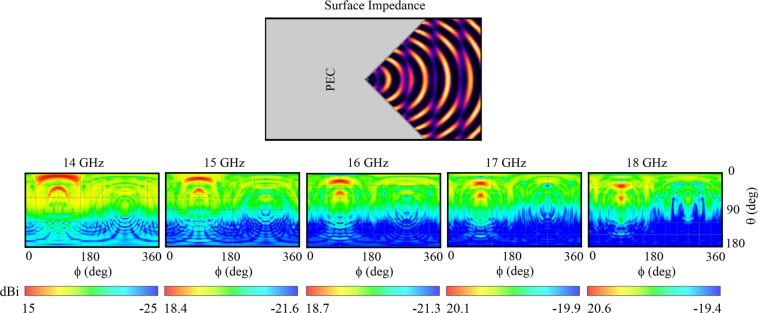
Surface impedance configuration of dual-beam generating metasurface and 2-D distributed radiation patterns of the wide-band dual-beam hologram over 14–18 GHz indicating elevation angle scanning. The simulation result shows two major lobes directed in desired angles at design frequency *f*_0_ = 18 GHz.

Substituting dual-beam object wave expression (19) in (1), the surface impedance needed for dual-beam generation can be obtained. Assuming that the object waves are directed in ${\theta }_{1}=30^\circ$ and ${\theta }_{2}=60^\circ$ in *z*–*y* plane (${\varphi }_{1}={\varphi }_{2}=90^\circ$), Fig. 9 shows the holographic surface impedance for such a hologram. The surface impedance is excited using cylindrical surface wave launcher and the reference wave should be updated after reflection from wedge reflector (reference wave expression is 14 again). Operation in wide frequency range was the result of the backward mode suppression for holograms producing single object wave. To illustrate whether the concept is generalizable to multiple object waves, simulations for further frequencies and elevation angle scan are done and can be seen in Fig. 9. Observe that the hologram still keeps scanning the elevation angle in *z*–*y* plane as the frequency varies. The difference in object waves’ angles does not change by frequency variation and preserves at 30° approximately.

Generating multiple object waves each directed in a direction without complicated feeding network is an attractive application of leaky wave metasurfaces^17,18^. Leaky wave multidirectional beamforming without holography is a complicated procedure. For instance, one approach is multi-beam pillbox leaky wave antenna based on SIW technology^29^. The design provides scannability property by frequency variation from 23.5 GHz to 26 GHz with fractional bandwidth of 10%. However, the proposed reflector-enabled hologram can achieve single-beam frequency scanning between 13–19 GHz (representing 37.5% fractional bandwidth) and multi-beam frequency scanning between 14–18 GHz (25% fractional bandwidth).

On the other hand, the conventional microwave holograms operate at a narrow frequency bandwidth. The Rabbit’s ears phenomenon cancellation using reflectors can help to obtain an acceptable operating frequency range for holographic multiple object waves beamforming.

## Experimental Validation

**Figure 10:**
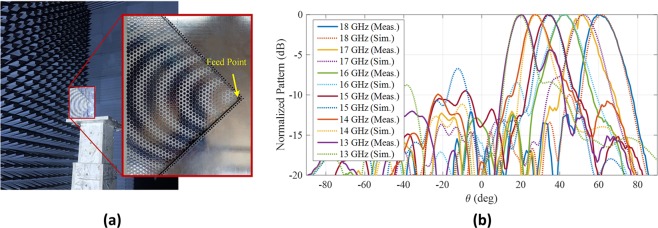
(**a**) Fabricated prototype of the hologram with wedge reflector. (**b**) Normalized radiation pattern of the proposed hologram with the wedge reflector at operating frequency range from 13 GHz to 18 GHz. Solid and dashed lines represent simulation and measurement results, respectively.

To experimentally validate the concept, the designed wedge reflector-supported hologram was fabricated which is shown in Fig. 10a. The surface wave launcher should be capable of generating Hankel surface wave over an acceptable frequency range. Among various types of surface wave launchers of this kind, the monopole antenna is a reasonable and simple choice. Since, fabrication of a PEC reflector is not possible using printed circuit board technologies, metalized via wholes are utilized to simplify the fabrication process. To realize the surface impedance, it has to be discretized and each sample is realized using hexagonal patch which represents the same surface reactance. The radiation pattern of the fabricated holographic antenna was measured in an anechoic chamber as shown in Fig. 10a. Figure 10b shows the simulation and measurement results for the radiation pattern over a frequency range of 13–18 GHz demonstrating an excellent agreement between the simulations and measurements. Both simulation and measurement results indicate the frequency-controlled beamscanning radiation provided by the proposed design.

## Conclusion

The Rabbit’s ears phenomenon is known for the destructive effect of the forward and backward modes in a center-fed hologram which severely restricts the bandwidth of the design to a single frequency. We proposed reflector-enabled holograms to suppress the backward mode and refine the surface wave distribution in a more constructive manner. We showed theoretically and experimentally that a wedge reflector-supported hologram provides beamforming and beamscanning features in a wide frequency bandwidth. Our simulation results demonstrated that this concept is not limited to single-beam holograms but can be generalized to design of multi-beam holograms. Two reflector shapes were designed to provide a comparison on reference wave distribution. Another discussion showed the wide-band acceptable operation of the hologram with wedge reflector versus some other types of holograms. The capability of scan by frequency variation is resulted for microwave 2-D holograms due to the acceptable operation frequency range. The fact that the forward mode dominant holographic reference wave provides wide frequency band and scannibility is also true for multi-beam holograms.
